# Human studies of anorectal sensory function

**DOI:** 10.1007/s11845-018-1847-5

**Published:** 2018-06-20

**Authors:** Charles H. Knowles

**Affiliations:** Blizard Institute, Barts and the London School of Medicine and Dentistry, Queen Mary University of London, London, UK

**Keywords:** Faecal incontinence, Human studies, Sacral neuromodulation

## Abstract

This review addresses three main questions: (1) Why is anorectal sensory function important in humans? (2) What is the evidence for anorectal sensory dysfunction in disease? (3) Can anorectal sensory function be modified for therapeutic benefit?

## Introduction

### Why is anorectal sensory function important in humans?

Humans have what is termed a social pattern of defaecation, i.e. one that delays bowel opening until a convenient moment and acceptable location, e.g. a toilet. This rather obvious remark has importance since it underpins the concept that humans require conscious perception of the need to defaecate with sufficient advanced warning such that social defaecation can be planned and executed. This function has evolved in humans and also exists in primates and other mammals that live in closed quarters, e.g. dogs, cats and even rodents (see James Jones review in this Festschrift). It is not present in more primitive eukaryotes, e.g. worms, nor, it would appear, in unconfined animals that graze, e.g. ungulates. This makes teleological sense in terms of spread of disease, etc.

Conscious perception implies sensory connectivity between the main organ of faecal storage (the rectum) and the brain. The detailed anatomy of the nervous system in respect of this function is incompletely understood in higher mammals and difficult to study in small experimental animals, e.g. rat and guinea pig because of translational differences, e.g. rodents have no specified division between colon and rectum. Nevertheless, the following points can be summarised:

- Afferent nerve endings relevant to defaecation reside not only in the rectum but also in the upper anal canal where they may ‘sample’ rectal contents [1]. In addition to relaying conscious discrimination of rectal contents, such endings are also probably critical to the reflex maintenance of continence (not covered in this review).
- Sensory endings must be able to not only detect distension (stretching or pressure) but also distinguish faecal contents (solid, liquid and air). The latter function underpins the ability to selectively detect and pass flatus and may be mediated by multimodal endings such as those responding to temperature and chemicals (taste) as well as pressure.
- Pressure and stretch receptors are located in the myenteric plexus as intraganglionic laminar endings (IGLEs) and signal via pelvic parasympathetic nerves [2, 3]. It is not known whether the muscularis propria also has intramuscular arrays (IMAs). Thermosensory and chemosensory functions are probably mediated by free nerve endings in the mucosa and submucosa and probably signal via spinal nerves. This organisation is in keeping with the much better studied upper GI tract where physiological functions such as proprioception are mainly mediated via vagal rather than spinal innervation.
- Somatic nerve endings contribute to distal rectal and upper anal sensory function via the pudendal nerve [4].

Regardless of the above, it is a fact that all sensory nerves must reach the spinal cord and thence the brain to sub-serve perception and these do so by following the vasculature in keeping with all other areas of the mesenteric innervation. Having crossed the mesorectum, such nerves all reach the posterior pelvis and pelvic side walls to join the dense plexus over the sacrum (the pudendal nerves lie below the pelvic floor and other nerves above). This anatomy is precarious in the sense that it is subject to compression and stretching of the pelvic floor and side walls such as occurs in pregnancy and childbirth (but also probably in prolonged straining and morbid obesity). The strong epidemiological link between pregnancy and childbirth and defaecatory disorders (especially faecal incontinence) is very well established [5] and has been shown to be in part mediated by nerve injury (with a focus on the pudendal nerves) [6, 7].

### What is the evidence for anorectal sensory dysfunction in disease?

There is clear evidence that disturbances of anorectal sensation are associated with a variety of diseases. This section will mainly focus on defaecatory disorders (constipation and faecal incontinence). Such evidence comes from three main sources:

#### Patient reporting

While this is often overlooked in favour of objective measurements, it is well known that many patients readily volunteer symptoms that attest to loss or gain in sensory perception. In patients with chronic constipation, it is not uncommon to find patients with no spontaneous urge to defaecate. Such patients are dependent thence on digitally assessing the rectum and aiding evacuation with manual measures or enemas; this deficiency is embodied within the ‘spontaneous complete bowel movement’ outcome measure used in some major pharmacological trials [8]. Other patients, for instance those with faecal incontinence, have a late or extreme urge to defaecate. Such ‘urgency’ may lead to frank incontinence if toilet facilities are distant. The time between first urge and involuntary passage of stool can be recorded as the deferment time and may be reduced to a few minutes in patients with faecal incontinence (FI) in contrast to healthy adults where (in the absence of acute diarrhoeal illness) most can defer days if necessary [9]. Finally, many patients describe an abnormal urge to defaecate with feelings of abdominal cramps rather than a pressure or fullness in the pelvis. An instrument to record five sequential defaecations in terms of visceral sensation has been recently developed and shows such differences between disease and health in adults and children (http://www.gastrojournal.org/article/S0016-5085(16)33164-X/abstract).

#### Standard (clinical) tests of anorectal sensation

The principle of distending the rectum to evoke perception of filling was first introduced by Goligher and Hughes in 1951 [10]. Studies of disease have demonstrated rectal hypersensitivity in patients with IBS in 1973 [11] and colitis in 1978 [12]. Subsequent studies of patients with constipation and incontinence show a proportion of patients with blunted sensation (rectal hyposensation) [13–15]. A plethora of further studies have confirmed these findings, especially in IBS where visceral hypersensitivity is now considered a biomarker of the condition.

Methodologically, rectal distension can be performed using a simple syringe and balloon catheter assembly [15] or a electromechanical barostat [16]. A full discussion of the pros and cons of these two approaches can be found elsewhere [17]. In brief, both depend on evaluating the patient’s threshold for first constant sensation, defaecatory desire and maximum toleration. Barometric distension is considered more valid and yields additional variables such as compliance of the rectal wall. It does so however at considerable cost in terms of investigation time, patient acceptability and complexity of protocols (ramp vs. phasic distension; methods of levels or limits). While a new ‘rapid barostat’ may change practice, most specialist centres currently employ volumetric distension only or reserve barostat protocols for specific patients. A further issue of both methods is the delineation of normality with little international consensus on the relative significance of single or multiple threshold abnormalities. Nevertheless, Table 1 summarises data using rectal sensory testing in main patient groups.

**Table 1:** Summary of rectal sensory findings in disease based on summation of data from published literature using a variety of methods (see text)

Condition	Hypersensitivity	Hyposensitivity
Irritable bowel syndrome	40–90%	15%
Chronic constipation	5%	25%
Faecal incontinence	30%	10%

#### Advanced (research) tests of anorectal sensory pathways

In this Festschrift collection, Professor Jones’ preceding review introduced an animal model in which somatosensory-evoked (using anal electrical stimulation) brain activity could be disrupted by pelvic nerve compression injury. In patients with FI, a small number of electrophysiological studies have suggested blunting of anal electrical sensation [18]. Also, prolongation of evoked potential latencies has been demonstrated using pudendal electrical stimulation [19] or rapid balloon rectal and anal distensions [20]. Collaborative studies of patients with FI from our own group (QMUL) and UCD demonstrate a disruption in somatosensory pathways from the anal canal that is almost identical to that observed in our rodent model of pelvic nerve injury. Interestingly, this finding was limited to parous females [*unpublished data*]. In patients with chronic constipation and rectal hyposensitivity, prolongation of latencies to rectal (electrical) evoked potentials has also been demonstrated [21].

In parallel, dynamic brain imaging techniques have been applied extensively to visceral evoked activities in patients with IBS using either PET or fMRI [22]. No studies of similar quality have yet been performed in patients with incontinence or constipation.

### Can anorectal sensory function be modified for therapeutic benefit?

The principle of modulating anal or rectal sensory functions for therapeutic benefit is well engrained by reconditioning techniques such as biofeedback where sensory training can be used to recognise smaller distensions and/or to re-coordinate rectal contraction concomitant with sensation of rectal filling [17]. Aside from this, the medical (non-surgical) literature is mainly limited to the suppression of visceral hypersensitivity in patients with abdominal pain, especially in relation to IBS [23]. There is currently no established medical therapy for treating visceral hyposensitivity, and remarkably, it remains unclear whether other drugs, for instance opioids, can induce rectal hyposensitivity despite their obvious role in inducing conditions associated with blunted rectal sensation, i.e. chronic constipation.

In contrast, the surgery literature is well served by studies that have investigated the effect of sacral neuromodulation (SNM) on anorectal sensory function. SNM is now the first line surgical intervention for patients failing non-surgical treatments for FI. Relatively early on in the evolving literature concerning SNM efficacy, reports of restoration of patient self-reported sensory functions, and increased deferment times [24] were augmented by observations of improved (more normalised) sensory thresholds to balloon distensions [25]. Many studies now confirm this observation using both volumetric and barostatic methods [36]. These include two small double-blind randomised trials performed during the percutaneous nerve evaluation stage of SNM [26, 27]. Indeed, restoration of rectal sensory function is the only physiological effect to have been independently confirmed in randomised blinded study designs and accords with urological (bladder) literature derived from both experimental animals and man [28, 29].

The effect of SNM on normalising (anal) somatosensory evoked potentials in a rodent model of nerve injury is covered in the review by James Jones (this series). In humans, SNM was found to cause a small though statistically significant reduction in latency using pudendal electrical stimulation [19] A paucity of data also exist for anal sensation [20] and for brain imaging (http://www.isrctn.com/ISRCTN98760715). A large NIHR-funded European trial of SNM efficacy and mechanism (including UCD) [the SUBSONiC study] will use magneticoencepalography [collaborating with the Aston Brain Centre] to study patients with FI undergoing SNM or sham stimulation to resolve both temporal and spatial resolution of cortical and subcortical responses alongside changes in symptoms [30].

These observations are not simply an intellectual curiosity. SNM, although currently pre-eminent as a safe and effective treatment for adults with severe FI, is not a panacea. It is recognised that more accurate prediction of responders vs. non-responders would allay ongoing concerns regarding long-term effectiveness and cost. The problem of patient selection is highlighted by ‘indecision’ in recent international guidelines [31] where patients with evidence of sphincter injury can be considered suitable for sphincter repair or SNM. It is clear that patients with high-grade obstetric sphincter trauma and immediate onset of symptoms, and also those with a cloacal defect, should be offered some form of repair and perineal reconstruction. However, these form the small minority of patients in colorectal surgical practice. Rather, the majority are women who develop symptoms much later in life (after childbirth), e.g. in their 50s and 60s. A high proportion of such women will have some form of sphincter defect/deficiency/scarring based on imaging with endo-anal ultrasound [32] but this is probably a minor contributor (or even bystander) to their functional deficit based on epidemiological data [5, 33]. Rather, these patients have a pathophysiology whose main contributors are pudendal and pelvic autonomic neuropathy, as well as endo-pelvic fascial and ligamentous injuries. The latter are detectable by clinical examination and imaging as pelvic organ prolapse syndromes and (when deemed significant) should be primarily corrected ahead of other interventions for FI [31]. The former however are difficult to detect reliably with routinely available tests (e.g. pudendal nerve terminal motor latencies). That is, unless one considers anorectal sensory testing as a marker of global pelvic nerve injury, which might not only detract the surgeon from performing a sphincter repair but also push decision-making toward SNM (based on above data). While difficult to prove definitively (this would require a trial with a stratified medicine design), some thought should also be given to whether this brings back into consideration (after failure of two RCTs to show benefit of SNM over sham stimulation in patients with chronic constipation in general [34, 35]) a group of patients (with rectal hyposensation) whose main symptoms are those of rectal evacuation rather than incontinence [26], i.e. a phenotype akin to ‘Fowler’s syndrome’ of the bladder (which responds preferentially to SNM [27]).

In summary, anorectal sensory function is important in humans and is evidenced by both clinical observations and specialist objective tests. While research is ongoing, it would appear that anorectal sensory function is modifiable in association with therapeutic benefit and may function as a future stratifier for clinical decision making.
